# COVID-19 vaccines: resolving deployment challenges

**DOI:** 10.2471/BLT.21.020321

**Published:** 2021-03-01

## Abstract

After the record-breaking pace of vaccine development, vaccine roll-outs are getting off to a frustratingly slow start. Tatum Anderson reports.

For Dr Dace Zavadska the coronavirus disease 2019 (COVID-19) vaccine roll-out began with a shipment of the Pfizer/BioNTech vaccine. The shipment arrived on 26 December 2020 in two boxes containing a total of 9750 doses.

“It was like a Christmas present,” says Zavadska, an infectious disease specialist, who is head of the vaccination centre at the Children’s Clinical University Hospital in Riga, the capital of Latvia.

Zavadska and colleagues in Riga’s other two university hospitals opened the present and got to work.

“It was slow at first because the vaccine requires careful handling, and we needed to persuade people that the vaccine was safe,” Zavadska says, “but by the end of the second day each nurse was vaccinating around eight patients per hour.”

Despite the efforts of health workers, by the end of the first week of January 2021 only 23 400 doses of the Pfizer vaccine had been dispensed, a rate judged to be too slow by Prime Minister Arturs Krišjānis Kariņš.

According to Louise Henaff, a technical officer at the World Health Organization (WHO) working on country preparation for COVID-19 vaccines roll-out, Latvia is not the only country getting off to a slow start.

“The roll-out has generally been slow,” she says, citing the example of European Union countries (of which Latvia is one) where only around 2% of the population (9 million of 448 million) had been vaccinated by the end of January.

A recurrent issue has been the requirement for cold chain storage, the Pfizer vaccine must be stored at around –70 °C, a good deal colder than the –20 °C achieved by most freezers, including those used in hospitals and clinics.

“We really wanted to jumpstart preparations.”

Ann Lindstrand

According to Zavadska, the lack of ultra-cold freezers was one of the main reasons Latvia did not take more of the Pfizer/BioNTech vaccine in the first weeks of the roll-out. “We had ultra-cold freezers at the central blood bank in Riga and in some of our bigger hospitals, but beyond that our capacity [to store the vaccine] was and still is limited,” she says.

Other countries face similar challenges. In Sri Lanka, for example, the Ministry of Health conducted a resource-mapping exercise in November 2020 and found that the National Immunization Programme had no ultra-cold freezers. However, like Latvia, Sri Lanka does have some capacity in blood banks.

“The authorities discussed investing in cold-chain infrastructure development, but they also considered alternative vaccines,” says Dr Deepa Gamage, a consultant medical epidemiologist at the Ministry of Health in Colombo.

Mustering the requisite human resources to implement national vaccination campaigns is also a concern. Because most countries are prioritizing the vaccination of older people, health workers at high risk of exposure to the virus and other people deemed to be particularly at risk, they are having to adapt their vaccine delivery systems.

Higher income countries are seeking to leverage capacity developed to implement seasonal influenza vaccine campaigns which typically target older people. This is true, for example, of Latvia, where the health ministry is co-opting health workers who usually administer the annual influenza vaccine.

Latvia is using information from municipalities to identify vulnerable groups, including people who may not be able to leave their homes, so that the vaccines can be taken to them. According to Zavadska, health workers are being trained to administer the new vaccines via online meetings and webinars.

Unfortunately, very few low- and middle-income countries have annual influenza vaccination programmes and are thus building on existing capacity in their routine child immunization programmes to deliver the COVID-19 vaccines. Sri Lanka is one of them and, according to Gamage, the health ministry will likely be relying on the country’s primary health-care clinics to deliver the shots.

“Nurses and midwives will be the main groups delivering injections in the clinics, but because of the sheer volume of vaccinations required and the tight delivery schedule, the authorities will also likely be drawing on the cadre of public health inspectors who usually administer human papillomavirus vaccines in schools,” Gamage says.

Where basic primary health-care coverage is lacking, governments will be looking to work with private sector providers. Dr Claudia Vivas Torrealba of the Health Systems Strengthening unit at the United Nations Children’s Fund (UNICEF) is already seeing governments investigating a mix of public and private health-care provision. “Many are considering geo-mapping of private sector providers to identify potential collaborations,” she says.

“At least we now know there is a light at the end of the tunnel.”

Dace Zavadska

Tapping into community resources is also going to play a key role, especially where primary health-care coverage is limited. In Cambodia, for example, health authorities already rely on an army of vaccinators, who deliver vaccine supplies on motorbikes or sometimes on foot to villages situated too far from primary health-care clinics.

According to Jalaa’ Abdelwahab, deputy representative for UNICEF in Cambodia, it is going to be particularly difficult to get cold vaccines to remote villages. “There are just two main airports in Cambodia so vaccines transported by road would require refrigeration for journeys that can take 12 hours,” he says.

A 20-year veteran of polio eradication campaigns, Abdelwahab is acutely conscious of the importance of reaching everyone. “Experience with other infectious disease pandemics, including polio, shows that the most marginalized communities end up being the last reservoirs for pathogens when we fail to get vaccines and other basic services to them,” he says.

According to Dr Ann Lindstrand, a vaccines expert with WHO’s Essential Programme on Immunization (EPI), WHO is supporting countries’ efforts to muster available resources, working in collaboration with UNICEF, the World Bank and other partners. “Efforts are being focused on an initiative designed to assist 100 countries create national deployment and vaccination plans in 100 days,” she says. “Because of the size of the roll-out and the speed with which it is being implemented, we really wanted to jumpstart preparations.”

Countries participating in the initiative apply the Vaccine Readiness Assessment Tool that WHO published in September 2020 to help countries identify strengths and weaknesses. In the European Region, WHO is working with governments using a UNICEF supply chain assessment tool that enables health and other government officials to review the performance of 13 critical operational and technical supply chain functions.

WHO is also encouraging the establishment of expert panels, typically referred to as national immunization technical advisory groups (NITAGs) to make sure plans are evidence based.

“In many countries, NITAGs are already in place to support the development and implementation of childhood vaccination programmes,” says Henaff, who hopes they can play a key role in optimizing the roll-out of COVID-19 vaccines.

However, she recognizes the immense challenges they face. “NITAGs are having to provide recommendations on vaccines for which there is still limited evidence and target populations that are different from the usual childhood and adolescent programmes,” she says.

How far NITAGs will be able to fulfil their role will depend in part on how much they are listened to by high level government officials who, because of the particularly severe and cross-cutting health and socioeconomic challenges posed by COVID-19, have been drawn into policy debates and policy-making.

In the words of WHO’s Lindstrand: “Decision-making is being carried out at much higher levels – by presidents and prime ministers – than is usually the case with immunization campaigns.”

Many of the decisions being made reflect difficult trade-offs, including trade-offs with other immunization campaigns. This is true, for example, of measles. Preventive and responsive measles vaccination campaigns were either paused or postponed in 24 countries in 2020 as resources were directed towards COVID-19 responses.

For Professor Rudzani Muloiwa, head of the Department for Paediatric and Child Health at the University of Cape Town in South Africa, and a member of South Africa’s NITAG, all-out efforts to implement COVID-19 vaccination risks undermining other essential immunization campaigns, especially if health workers and other resources are co-opted to immunize adults.

“People are already bracing themselves for measles outbreaks because of the negative impact of COVID-19 on EPI vaccination coverage over the last year,” he says.

In the coming months, national governments worldwide – or at least those lucky enough to procure COVID-19 vaccines – will be wrestling with many difficult trade-offs. Whatever decisions they come to, it will be down to dedicated health workers to make sure that the vaccines get into patients’ arms. Health worker Dace Zavadska says she and her team vaccinate from eight A.M. to six P.M. without a break. “It is really hard work,” she says, “but at least we now know there is a light at the end of the tunnel.”

**Figure:**
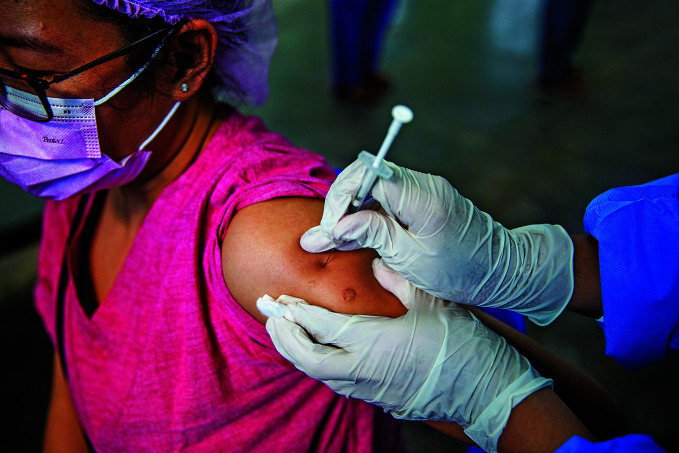
A health worker is vaccinated against COVID-19 at Yangon General Hospital, in Yangon, Myanmar.

UNICEF/Nyan Zay Htet

**Figure:**
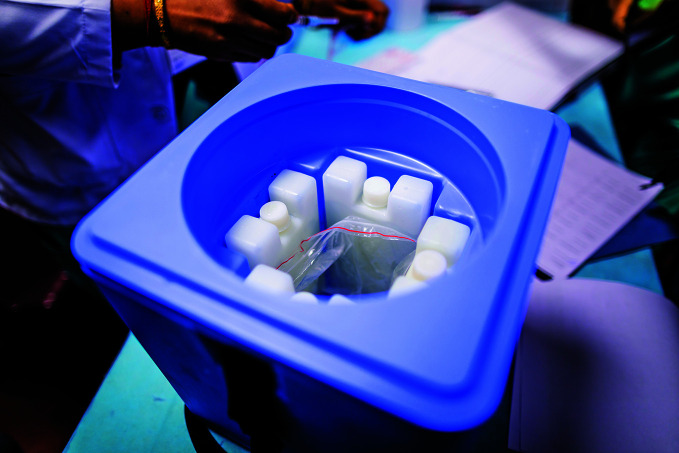
Cold storage of COVID-19 vaccine at Pt. Madan Mohan Malviya Hospital in New Delhi, Delhi, India.

UNICEF/Sujay Reddy

